# A new taxonomy for perceptual filling-in

**DOI:** 10.1016/j.brainresrev.2010.10.004

**Published:** 2011-06-24

**Authors:** Rimona S. Weil, Geraint Rees

**Affiliations:** Wellcome Trust Centre for Neuroimaging, University College London, 12 Queen Square, London WC1N 3BG, UK; Insitute of Cognitive Neuroscience, University College London, 17 Queen Square, London WC1N 3AR, UK

**Keywords:** Filling-in, Perceptual completion, Vision

## Abstract

Perceptual filling-in occurs when structures of the visual system interpolate information across regions of visual space where that information is physically absent. It is a ubiquitous and heterogeneous phenomenon, which takes place in different forms almost every time we view the world around us, such as when objects are occluded by other objects or when they fall behind the blind spot. Yet, to date, there is no clear framework for relating these various forms of perceptual filling-in. Similarly, whether these and other forms of filling-in share common mechanisms is not yet known. Here we present a new taxonomy to categorize the different forms of perceptual filling-in. We then examine experimental evidence for the processes involved in each type of perceptual filling-in. Finally, we use established theories of general surface perception to show how contextualizing filling-in using this framework broadens our understanding of the possible shared mechanisms underlying perceptual filling-in. In particular, we consider the importance of the presence of boundaries in determining the phenomenal experience of perceptual filling-in.

## Research Highlights

► We propose a new taxonomy for perceptual filling-in. ► We review experimental evidence for different forms of filling-in. ► We show how this new framework sheds light on mechanisms for perceptual filling-in. ► We consider the importance of boundaries in perceptual filling-in.

## 1 Introduction

Perceptual filling-in is the interpolation of missing information across visual space. It is a ubiquitous process in the central visual system, necessary to make sense of the world. Light from portions of objects and scenes often falls upon parts of the retina without photoreceptors, such as the blind spot or retinal vessels, or falls behind objects in the real world and the visual system must process information across these occluders so that they are perceived as complete and not fragments. It is an extremely effective process, as most of the time, we are entirely unaware that it is taking place. It is also a natural process that takes place all the time, but researchers have observed that certain stimuli can be configured to promote perceptual filling-in. These may exploit some of the same mechanisms underlying other forms of filling-in.

Perceptual filling-in is a heterogeneous phenomenon. Here we discuss different forms of filling-in and present a new framework for categorizing the various types of perceptual filling-in. We propose that this system, by grouping together common aspects of filling-in and separating out contrasting features, may provide some insights into possible mechanisms underlying the different forms of perceptual filling-in.

### 1.1 Nomenclature of perceptual filling-in

There is some confusion in the literature regarding nomenclature, with the terms filling-in, perceptual filling-in, and perceptual completion all being used to describe the interpolation of missing information across visual space but sometimes for different forms of filling-in. In particular, some groups use filling-in to refer to surfaces (Sasaki and Watanabe, 2004) and perceptual completion to refer to contours (Sergent, 1988), but other groups use the terms interchangeably (Pessoa et al., 1998, De Weerd, 2006). Here we use the terms perceptual completion and perceptual filling-in interchangeably to refer to the perceptual experience of interpolation of information across visual space, for both contours and surfaces. It is also important to distinguish perceptual filling-in from neural filling-in (Pessoa et al., 1998). We use perceptual filling-in here to refer to the perceptual experience of interpolation and do not imply anything about neural mechanisms. Where mechanisms are being considered, this will be explicitly stated in the text. We also restrict the use of the term perceptual filling-in to situations where there is a conscious experience of the information that is filled-in, rather than other forms of gleaning absent information such as awareness of the space behind the head. Filling-in is also used in areas of visual neuroscience to refer to aspects of surface perception (Neumann et al., 2001). Although perceptual filling-in may share some of the same mechanisms that are involved in normal surface perception, this is not specifically being explored here.

### 1.2 Current theories of mechanisms of perceptual filling-in

Two main models have been proposed in the literature to account for perceptual filling-in. One model proposes that neural filling-in does not occur but that structures of the visual system simply ignore the absence of information across the scotoma or blind spot and label the region with the information in the surround (“more of the same”) (Dennett, 1991, Kingdom and Moulden, 1988, O'Regan, 1992). According to this theory, also termed the symbolic or cognitive theory, there would be no retinotopic representation of the filled-in surface in earlier visual regions, but activity related to filling-in might be found in higher visual areas representing objects.

However, substantial evidence is accumulating for an active process for some forms of perceptual filling-in, with point-for-point representations of filled-in regions in retinotopic cortex (Fiorani et al., 1992, Matsumoto and Komatsu, 2005, Tong and Engel, 2001, Redies et al., 1986). This second model, known as the “isomorphic model”, might be mediated by lateral propagation of neural signals, with the spread of activation across the retinotopic map from the border to interior surface of the filled-in figure (De Weerd et al., 1995). An alternative mechanism might be passive remapping of receptive fields (Chino et al., 2001) such that visual input from the region surrounding the scotoma or blind spot is displaced so that it infiltrates the cortical region representing the blind spot or scotoma.

### 1.3 Previous taxonomies of perceptual filling-in

Taxonomies of perceptual filling-in have been previously proposed. Pessoa et al. (1998) proposed two general divisions: modal versus amodal completion and boundary versus featural completion. However, this taxonomy has been criticized as being unnecessarily complicated (Birgitta Dresp, 1998, commentary on Pessoa et al., 1998) and the major division of modal/amodal as unhelpful (Carol Yin, 1998, commentary on Pessoa et al., 1998).

Dresp proposed a taxonomy based purely on psychophysical evidence rather than phenomenology. She proposed divisions based on area, surface, and contour completion, where area completion involves spreading of contrast into regions without clearly defined boundaries; surface completion involves the perception of figures from real or apparent contours, and contour completion involves the perceptual grouping of collinear lines. However, it is not clear that the distinction between area and surface completion is helpful and dismissing all phenomenological differences between types of perceptual filling-in misses the aim of a framework to explore commonalties and differences to better understand underlying mechanisms.

Yin proposed a taxonomy reflecting the goals of visual completion: unity, shape, and perceptual quality. However, this taxonomy is very similar to the original Pessoa et al. taxonomy (with shape determination closely linked to boundary completion and perceptual quality determination linked to featural completion) and it has been suggested that unity does not necessarily involve perceptual completion as disparate fragments may be experienced as unified without any visual completion taking place (for example, when a group of dots moving coherently are experienced as unified) (Pessoa et al., 1998).

### 1.4 A new framework for categorizing different forms of perceptual filling-in

Here we propose a new framework for perceptual filling-in based on phenomenological and psychophysical characteristics. We suggest that this framework provides some insight into the possible underlying processes involved in filling-in by grouping the various types of perceptual completion.

Perceptual filling-in can be broadly separated on phenomenological and psychophysical grounds into two types: perceptual filling-in that takes place instantly and perceptual filling-in that is delayed as it requires prolonged fixation before it can occur. These can each be further subdivided according to whether perceptual filling-in will occur only in the presence of specific stimulus configurations (stimulus-dependent) or will occur regardless of stimulus configuration (stimulus-independent, often due to an intrinsic visual system phenomenon, see Table 1). We will now explore the current understanding of perceptual filling-in within these categories and consider how this categorization helps understanding of the processes involved in perceptual filling-in. This review is not intended to be exhaustive but will provide examples for each type of perceptual filling-in (instant versus delayed; stimulus-dependent and stimulus-independent) where these help to illustrate common and distinct features of filling-in.

**Table 1:** Framework for categorizing different types of perceptual filling-in, according to whether perceptual filling-in occurs instantly or is delayed and only occurs after prolonged fixation, and according to whether perceptual filling-in occurs only with specific stimulus configurations or will occur independent of the particular stimulus used.

	Stimulus-dependent	Stimulus-independent
Instant	Illusory contours	Filling-in at the blind spot
	Illusory surfaces	Filling-in of retinal scotomas
	Neon color spreading	
	Craik–Cornsweet–O'Brien effect	
	Afterimage color filling-in	
	Amodal completion	
Delayed	Troxler fading	Stabilized retinal images
	Artificial scotomas	
	Motion-induced blindness	

## 2 Instant perceptual filling-in dependent on stimulus configuration

### 2.1 Illusory contours

**Fig. 1:**
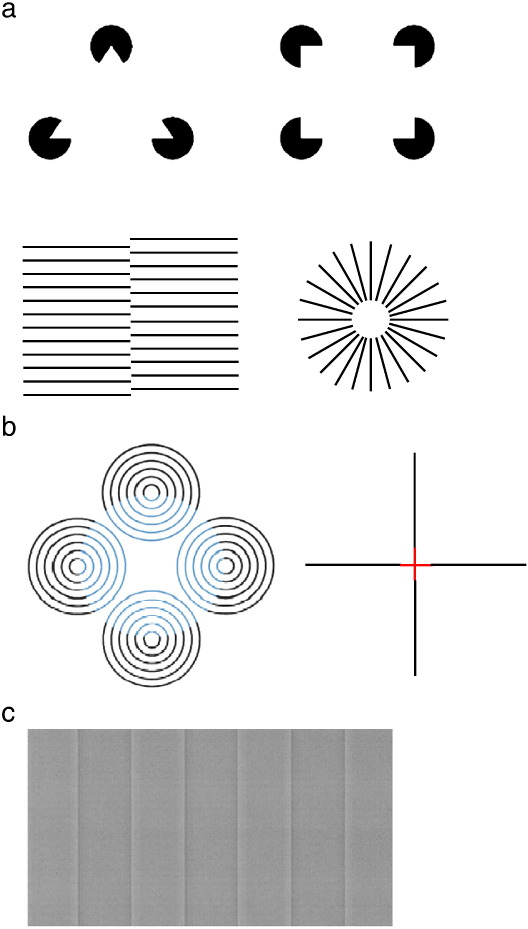
(a) Kanizsa figures and other examples of illusory contours. (b) Neon color spreading. (Left panel) The central virtual circle appears as a transparent blue surface overlapping the black rings. Adapted from Pinna and Grossberg (2005), with permission from the Optical Society of America. (Right panel) The Ehrenstein figure: red color from the inner red segments of the cross seems to leak into its surround to produce the impression of a central red disc. (c) A Craik–Cornsweet–O'Brien effect grating. Note that although the luminance in the central regions of each panel is identical, alternate bars appear darker or lighter due to differences at the edges of the bars.

One type of instant perceptual filling-in is the perceptual completion of illusory contours (also termed modal completion (Michotte et al., 1991)). These are edges that are perceived in the absence of physical boundaries. They are induced by an appropriate arrangement of local elements, or inducers, causing the perception of a surface overlaying these inducing elements. A classic example of illusory contours is the Kanizsa figure. This is generated by a particular configuration of high contrast figures, such as incomplete and co-aligned black circles, which induce the illusory perception of a light shape (Kanizsa, 1979) (see Fig. 1a).

Illusory contours can also be generated by displaced gratings, which can form edges or circular boundaries, depending on the configuration of the inducing gratings (see Fig. 1a) and can even be defined by depth cues (Mendola et al., 1999). This type of perceptual completion, in addition to inducing the appearance of a complete contour or shape, is also characterized by the striking phenomenon that the region intervening between the inducers is filled-in with illusory brightness and color that is determined by the inducers (Davis and Driver, 2003).

One area of debate is whether illusory contours are processed at the same anatomical level as real contours. Purely psychophysical studies in normal human vision suggest a locus of perceptual completion in earlier visual areas as the tilt after-effect and motion after-effects can be generated with illusory as well as real contours (Smith and Over, 1975) and tilt after-effects cross over between illusory and real contours (Berkley et al., 1994). Furthermore, Kanizsa figures are detected pre-attentively in a visual search paradigm (Davis and Driver, 1994), and if the high contrast inducers are placed on a checkerboard background that is misaligned with the inducing elements, the illusory contour disappears (Ramachandran et al., 1994), consistent with a close interaction between illusory contour processing and processes extracting local feature information. Moreover, perceptual completion of illusory contours is worse across the vertical meridian (Pillow and Rubin, 2002), reflecting limitations in cross-hemispheric integration and therefore a locus more likely to be in V1 or V2, areas that are more sensitive to this hemispheric divide. Finally, patients with visuospatial neglect show improved performance on line bisection tasks when illusory contours are present, despite being unaware of their presence (Mattingley et al., 1997) consistent with a preattentive locus of boundary completion.

Electrophysiological studies in animals suggest a locus for perception of illusory contours early in the cortical visual pathway (for a review, see Nieder, 2002). An illusory bar induced by aligned corners placed outside the classical receptive field of V2 neurons yields significant responses (Peterhans and Von der Heydt, 1989), and similarly, neurons in monkey V2 but not V1 responded to contours generated by abutting gratings (Von der Heydt and Peterhans, 1989). Subsequent experiments in cats have shown that both V1 and V2 neurons carry signals related to illusory contours generated by abutting gratings, although signals in V2 are more robust than those in V1 (Redies et al., 1986, Sheth et al., 1996). Furthermore, a recent study suggests the presence of interactions between V1 and V2 neurons in processing illusory contours associated with Kanizsa figures, with responses occurring earlier in V2 (70–95 ms) than in V1 (100–200 ms) (Lee and Nguyen, 2001).

A recent electrophysiological study in non-human primates seemed to implicate higher visual areas such as inferotemporal cortex (which may be homologous to human lateral occipital complex (LOC)), in perception of illusory figures generated by abutting gratings (Sary et al., 2007). However, that study examined only responses in inferotemporal cells (and not responses in V1 or V2 neurons) and showed little difference in responses between silhouettes of images and abutting gratings forming images. It is thus difficult to be sure whether the measured responses reflected detection of the images themselves, rather than a specific response to illusory contours.

Interestingly, surgical lesions to monkey inferotemporal cortex cause deficits in discrimination of figures generated by abutting lines, but no deficits in shape detection (Huxlin et al., 2000). This may suggest involvement of higher tier visual areas in perception of illusory figures produced by abutting lines but would still be consistent with a model of illusory contour formation earlier in the visual pathway and interaction with these higher areas. Moreover, local neurochemical changes surrounding the lesion may have wider effects on visual processing and can make lesion studies difficult to interpret.

Taken together, studies in animals suggest that illusory contour perception occurs in V2, with feedback to V1 and possible involvement of inferotemporal regions, particularly when the illusory contours generate shapes or images.

Illusory contours have been extensively studied in human neuroimaging experiments with conflicting results, some of which may be explained by confounding methodological issues, particularly differences in stimuli, such as contours generated by abutting gratings or by Kanizsa figures. Even for the same illusory contour type, differences such as stimulus size, ratio between inducers and total length of illusory figure, and different types of inducers, such as static and moving features, can be the cause of important confounds (for a review, see Seghier and Vuilleumier, 2006).

Illusory contours induced by abutting gratings are associated with specific activations within area V1 (Larsson et al., 1999, Mendola et al., 1999), and the timing of responses seen in MEG studies is consistent with involvement of early cortical visual areas (Ohtani et al., 2002). Similarly, some studies of illusory contours induced by Kanizsa-type figures also show activations within V1 (Seghier et al., 2000, Maertens et al., 2008) with similar early electrophysiological responses (Proverbio and Zani, 2002). However, other studies of illusory contours generated by Kanizsa-type figures do not show involvement of V1 during perception of illusory contours (Kruggel et al., 2001, Stanley and Rubin, 2003).

As with neurophysiological studies on primates, the involvement of area V2 in illusory contour perception has been more consistently shown, with most neuroimaging studies demonstrating activation of this region for both abutting line gratings (Larsson et al., 1999) and Kanizsa figures (Hirsch et al., 1995), although one study that examined responses to both types of illusory figure did not find reliable evidence for involvement of V2 for Kanizsa figures, despite strong responses in V2 for illusory contours generated by displaced gratings (Mendola et al., 1999).

Higher level regions have also been implicated in illusory contour processing. One fMRI study examining responses to illusory contours induced by both Kanizsa figures and figures generated by displaced gratings found activation specifically associated with illusory contour processing in putative Areas V7 and V8 and overlapping with the lateral occipital complex (Mendola et al., 1999). A similar region was identified in other studies investigating Kanizsa figures (Murray et al., 2002, Stanley and Rubin, 2003, Murray et al., 2004) and using ERP source mapping (Murray et al., 2004). It is possible that this area is activated partly due to object processing within the Kanizsa figure. Interestingly, an MEG study using line gratings but no illusory figure failed to detect activity within the LOC (Ohtani et al., 2002) and when illusory contour formation was prevented using misaligned inducers (Stanley and Rubin, 2003), LOC was still activated, suggesting that LOC activity may not be specific to illusory contour processing.

Other higher tier visual areas that have been implicated in processing illusory contours induced by Kanizsa figures include the right fusiform gyrus (Halgren et al., 2003) (also activated by illusory figures induced by displaced gratings (Larsson et al., 1999)) and the kinetic occipital sulcus (Seghier et al., 2000; Kruggel et al., 2001) and Areas VO1 and V3A/B (as well as LO1 and LO2) have been implicated in responses to displaced gratings (Montaser-Kouhsari et al., 2007).

Taken together, electrophysiological and neuroimaging studies suggest that illusory contour processing is associated with activity of neuronal populations in early visual areas, especially V2, and may also involve more global, high-level processing in higher visual areas, although higher visual areas seem to be more consistently involved when the illusory contours generate shapes and figures.

### 2.2 Illusory surfaces

#### 2.2.1 Neon color spreading

Neon color spreading is a striking and beautiful phenomenon whereby the neon-like glow of a color escapes the boundaries of a real figure and seems to fill the surrounding area until it is limited by the boundaries of an illusory figure (for a review, see Bressan et al., 1997) (see Fig. 1b).

This effect, first observed by Dario Varin in 1971 and Harrie van Tuijl (1975), also occurs for achromatic figures, with illusory brightness spreading induced by grey segments on black inducers. The illusion is strongest when the lines and segments are continuous, collinear, and equally thick and the effect disappears if the subjective figure is encircled by a ring (Redies and Spillman, 1982). Others have shown that depth cues are important in producing this effect, with neon spreading only occurring if the colored elements are phenomenally in front of the inducing areas (Nakayama et al., 1990). The rules for whether color or brightness spreading will take place seem to represent the color and luminance prerequisites for perceiving a transparent subjective figure, as spreading usually occurs when the luminance of the segments is between the luminance of the embedding lines and that of the background (Bressan et al., 1997).

Illusory contours seem to play a crucial role in delimiting the spread of neon color illusions, with spreading particularly vivid when colored segments are inserted into the blank area of a figure that otherwise would produce a colorless illusory figure (Watanabe and Takeichi, 1990).

Neon color spreading seems to involve very early visual processes: if the colored cross and black arms are presented to different eyes but aligned to form a fused image, an illusory contour is perceived, but no color spreading occurs (Takeichi et al., 1992) and neon color filling-in (and the related but contrasting watercolor illusion) has recently been explained in terms of competition within boundary perception (Pinna and Grossberg, 2005). Specifically, boundaries of lower contrast edges might be weakened by spatial competition more than boundaries of higher contrast edges and this induces spreading of color across boundaries, producing the illusion. Moreover, in a recent neuroimaging study in humans, neon color spreading was associated with activity in V1, which was independent of attention (Sasaki and Watanabe, 2004), consistent with a process involving early visual regions.

#### 2.2.2 Craik–Cornsweet–O'Brien illusion

The Craik–Cornsweet–O'Brien (CCOB) effect occurs when regions of equal luminance are perceived to differ in brightness due to a luminance transition at the border between these regions (Craik, 1966, O'Brien, 1958, Cornsweet, 1970) (see Fig. 1c). For example, in Fig. 1c, the bars appear to alternate between light and dark despite the fact that the central portions of the bars are of equal luminance. Thus the impression is similar to a square-wave grating of alternating bars of uniform luminance. Notice that this effect is abolished if the edges are occluded.

Early theories proposed that the mechanism driving the CCOB effect included an abstract process whereby a label such as “brightness” is attached to one region, possibly at a later stage of visual processing, similar to the symbolic theory of filling-in. This is based on observations that the appearance of a square-wave grating is not significantly altered by removing the low spatial frequency components, particularly at low contrasts (Burr, 1987, Campbell et al., 1978). Thus there is an assumption by the visual system that images have a square-wave structure. An alternative hypothesis is that the CCOB effect is mediated by a form of diffuse filling-in of featural quality (Cohen and Grossberg, 1984), consistent with psychophysical studies showing that illusory brightness propagates at a fixed speed across the central visual field (Davey et al., 1998). However, more recent psychophysical observations challenge this theory by showing that introducing luminance noise into previously uniform areas does not significantly affect the illusion (Dakin and Bex, 2003). These authors suggest an alternative mechanism, supported by psychophysical modeling, in which the illusion is mediated via amplification of the weak low spatial frequency structure in an image to make it conform to the spatial frequency structure of natural scenes (see also Devinck et al., 2007). Specifically, the model proposes that the visual system assumes that the underlying spatial frequencies in an image are similar to those in natural images, with a drop in amplitude of the spatial frequency components as a function of the spatial frequency (1/*f*) and that the visual system reweights the spatial frequency channels to match the average spatial frequency spectrum of natural images.

In cats, brightness responses induced by the CCOB effect are seen in Area 18 and to a lesser extent in Area 17 (Hung et al., 2001), which is consistent with local processes propagating brightness information. More recently, the same group showed in macaque that V2 thin stripe regions are responsive to illusory changes in brightness induced by the CCOB effect (as well as true changes in luminance) whereas V1 blob regions only encode physical changes in brightness, suggesting that V1 responses reflect luminance properties signalled by local inputs, whereas V2 may confer higher order properties resulting from integration of non-local inputs (Roe et al., 2005).

In humans, fMRI has been used to probe cortical responses to CCOB-induced changes in brightness (Perna et al., 2005). Initially, a caudal region of the intraparietal sulcus and the lateral occipital sulcus were found to respond specifically to the CCOB illusion, with earlier visual areas, including V1, responding just as strongly to a line of matched contrast and detectability, rather than specifically to the brightness illusion. The authors concluded that V1 does not specifically encode illusory brightness, but that regions higher than V2 compute surface brightness. However, a recent study using high spatial resolution fMRI in humans to image early visual areas challenges these conclusions by showing that illusory changes in brightness induced by the CCOB effect can modulate the responses of neurons in the lateral geniculate nucleus (Anderson et al., 2009). Importantly, this recent study also showed that the magnitude of the CCOB effect depends on the size of the region enclosed by the inducing border, with a greater effect for smaller regions. This may explain the discrepancy in findings between this and the earlier study, in which a large image (~ 15 visual degrees) was used, which might not have induced as robust filling-in.

In summary, psychophysical and neuroimaging studies in humans suggest that the illusory brightness induced by the Craik–Cornsweet–O'Brien effect may be mediated by amplification of the low spatial frequency structure of an image to bring the image statistics in line with natural scenes, very early in the retino-geniculate visual pathway.

#### 2.2.3 Afterimage color filling-in

**Fig. 2:**
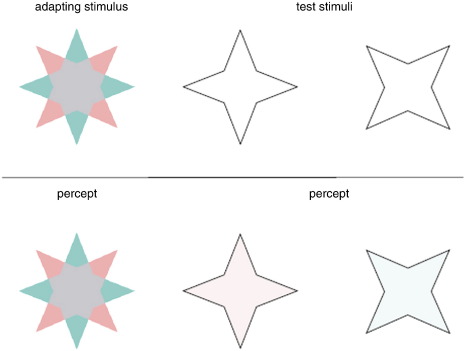
Illustration of color afterimages. After adapting to the adapting stimulus, the test outlines are presented alternately. The two outlines cause the appearance of a red and then a cyan afterimage, including in the center of the figure, where previously no color was present. , with permission from Elsevier.

Reprinted from van Lier R, Vergeer M, Anstis S: Filling-in afterimage colors between the lines. Curr Biol, 19 (2009), R323–R324.

A recent and striking perceptual filling-in phenomenon has been described (van Lier et al., 2009) whereby afterimage colors can spread to previously uncolored areas, when constrained by contours presented after the colored image (Fig. 2), demonstrating the important role of boundaries in constraining perceptual filling-in of afterimages.

Taken together, perceptual filling-in of surfaces seems to be closely linked to boundary perception (real or illusory) and is likely to entail a multilevel process involving higher regions feeding back to earlier visual regions.

### 2.3 Perceptual filling-in behind occluders: amodal completion

**Fig. 3:**
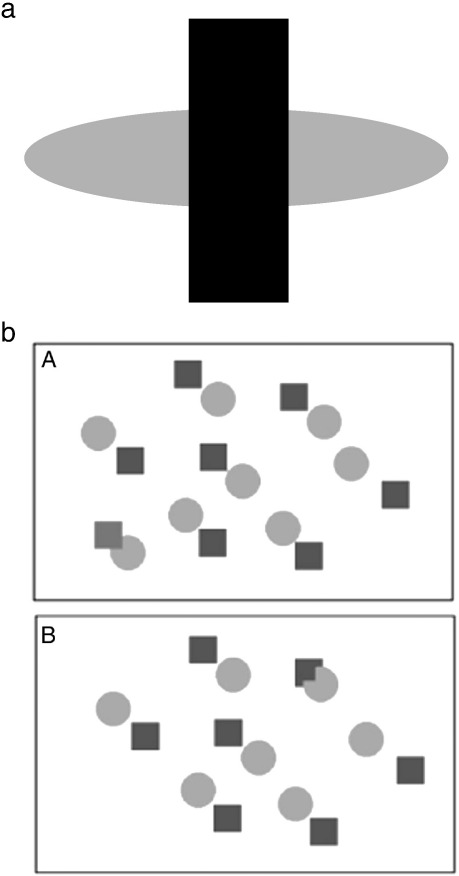
(a) Example of amodal completion. The appearance is of a grey oval behind a black rectangle, although the oval is not seen in full. (b) Visual search for amodally completed target. Identifying the notched circle is harder in panel a as it is amodally completed and perceived as a circle among the other circles. In panel b, the notched circle “pops out”. Search display is similar to those used in Rensink and Enns (1998), with permission from Elsevier. Adapted from Driver et al. (2001).

Objects in the natural world do not present themselves in isolation but are often occluded by other objects. Yet we do not have the impression of being surrounded by object fragments as the visual system perceptually fills in the missing information to interpret the objects as complete (Fig. 3a).

This type of perceptual filling-in, where fragments are taken to be the visible portions of an occluded object, is termed amodal completion. Several theories have been proposed to explain this linkage of image fragments. One classic theory is that local image cues are used to determine object relationships. For example, T-junctions are often present when an object is perceived as occluded and are likely to represent contour discontinuity (Kellman and Shipley, 1991, Nakayama et al., 1989). Another local cue is the relative orientation of image contours (Kellman and Shipley, 1991): objects are more likely to complete behind an occluder if the angle between their extensions behind the occluder is greater than 90^o^, such that good contour continuation is more likely. However, important exceptions can be shown where completion takes place in the absence of T-junctions or good contour continuation and conversely, where relatable edges are present but completion is not perceived (see (Boselie and Wouterlood, 1992, Tse, 1999) for examples).

Another theory emphasizes the importance of more global cues in causing perceptual completion, such as symmetry, regularity or simplicity (van Lier et al., 1994). According to this theory, completion tends to produce the impression of the most regular shapes (Buffart and Leeuwenberg, 1981). However, other researchers have shown that observers do not always perceive the most regular shapes (Boselie and Wouterlood, 1992). Other theories ascribe amodal completion to the use of volume cues to form the image (Tse, 1999) or common depth planes (Nakayama and Shimojo, 1992). Indeed, the presence of a depth-appropriate occluder plays a critical role in determining whether completion takes place (Johnson and Olshausen, 2005).

Amodal completion seems to take place early in visual processing. For example, when subjects are asked to search for a notched circle in an array of circles and squares, if the notched circle abuts the edge of a square so that it seems to be occluded by it, the notched circle takes longer to find (see Fig 3b) (Rensink and Enns, 1998) as it is perceived as a complete circle in a sea of complete circles.

Neurophysiological studies support the notion that amodal completion involves early visual processes, with evidence of amodal contour responses about 100 ms after presentation of occluded images in macaque V1 cells (Lee and Nguyen, 2001) and V1 monkey cells fire in response to an amodally completed bar behind a square (Sugita, 1999). V2 neurons in macaque also respond to partially occluded contours (Bakin et al., 2000). Moreover, in humans, ERP differences between occluded images and their deleted counterparts are seen as early as 130 ms after presentation (Johnson and Olshausen, 2005).

Initial neuroimaging studies seemed to contradict these findings with some studies showing increased activity in LOC for occluded compared to scrambled images with identical local features (Lerner et al., 2002). Yet the same authors found that completion effects occur very rapidly following stimulus onset (Lerner et al., 2004). More recently, fMRI adaptation paradigms have been used to show that the representation of an amodally completed figure evolves over time (Rauschenberger et al., 2006), with physical properties of the stimulus processed first (before 100 ms) followed by the amodal completion of the stimulus (within 250 ms). These findings were extended by a study identifying regions in early visual cortex involved in processing of local contour information during amodal completion and regions in inferior temporal cortex responding to the amodally completed shape (Weigelt et al., 2007) consistent with both local and global processing in amodal completion. Conversely, a recent study used a stereoscopic manipulation to reveal that LOC and dorsal object-selective foci are specifically responsive to completed occluded objects (Hegde et al., 2008).

Taken together, the process of filling-in behind occluders is likely to involve a large number of information processing steps in early and higher regions of visual cortex. These might involve distinguishing between the boundaries of the occluded and the occluding object, assigning each of the resulting partial views a surface and then filling-in the missing information of each part (Johnson and Olshausen, 2005) using clues from depth disparity and collinear edges and representing the fully completed shape.

#### 2.3.1 A common mechanism for modal and amodal completion?

A subject of intense debate is whether modal and amodal completion share a common mechanism as both involve the connection of disjoint image fragments into a coherent representation of an object, surface or contour. Kellman and Shipley (Kellman and Shipley, 1991) have argued in their ‘identity hypothesis’ that the same interpolation mechanism is responsible for both processes, but that the difference in appearance arises from depth of placement of completed contours and surfaces. However, others have shown that there may be differences in the mechanisms underlying modal and amodal completion. For example, Davis and Driver (Davis and Driver, 1994) found that search for modally completed figures is more efficient than for amodally completed counterparts and in a series of experiments, Anderson and colleagues (Anderson et al., 2002) provide evidence for different mechanisms for the two processes.

It is likely that modal and amodal completion share some common initial mechanisms within early retinotopic areas (Murray et al., 2004), and that these are followed by feedback processes within higher tier areas that modulate effects in the lower tier areas to generate the different percepts.

## 3 Instant perceptual filling-in independent of stimulus configuration

### 3.1 Filling-in at the blind spot

**Fig. 4:**
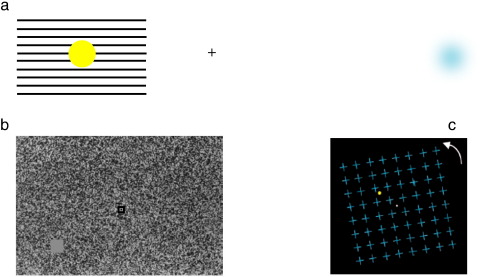
(a) (Left panel) Example of perceptual filling-in at the blind spot. Hold the page 15 cm from your face, fixate the cross, and close your right eye. When the yellow disc falls across the blind spot, it will seem to disappear and be perceptually filled-in by the horizontal bars. Adapted from Komatsu (2006), with permission from Macmillan Publishers Ltd.: *Nature Reviews Neuroscience*, 2006. (Right panel) Example of Troxler fading. Hold the page 20 cm away from your face, fixate the cross with both eyes open, after a few seconds, the blue pattern will fade and disappear. (b) Example of an artificial scotoma. A target is placed in the near periphery on the background of dynamic twinkling noise. Participants fixate centrally and the target gradually fades and disappears. Adapted from Ramachandran VS, Gregory RL: Perceptual filling in of artificially induced scotomas in human vision, Nature, 350 (1991), 699–702, with permission from Macmillan Publishers Ltd.: *Nature*, 1991. (c) Example of motion-induced blindness. The grid of blue crosses rotates and participants fixate the central white point while attending to the yellow disc. The yellow disc intermittently disappears and reappears (). Adapted from Schölvinck and Rees (2010).

The blind spot is the part of the retina where the optic nerve leaves the eye. It is devoid of photoreceptors and therefore carries no visual information from the corresponding region in visual space. It measures roughly 5^o^ in diameter and its center lies 15^o^ medial to the fovea, slightly above the horizontal meridian. In normal binocular vision, the cortical representation of the other eye compensates for this lack of visual information. Interestingly however, monocular viewing does not lead to the appearance of a blank patch in the visual field as the visual system perceptually fills-in visual information across the blind spot from the surrounding color and texture (Ramachandran, 1992). Importantly, filling-in at the blind spot occurs throughout normal monocular vision, but can be demonstrated using stimuli with particular configurations (Fig. 4a, left panel).

Behavioral studies suggest that this is an early, preattentive process involving sensory rather than cognitive mechanisms. If several rings are viewed, with one ring positioned over the blind spot, this will ‘pop out’ as it is perceived as a disc in a background of rings (Ramachandran, 1992)). Even an extremely narrow border (0.05 deg) surrounding the blind spot, will generate the appearance of uniform color filling-in the blind spot (Spillmann et al., 2006), consistent with the theory that this form of filling-in depends on local processes generated at the edge of the blind spot representation in early visual cortex. Studies also indicate that this is an active process, as the perception of filled-in motion at the blind spot causes a motion after-effect in the other eye suggesting that perceptual filling-in can cause adaptation of motion-sensitive neurons (Murakami, 1995). Perceptual filling-in at the blind spot induces little or no distortion of the surrounding region (Tripathy et al., 1996) which would argue against remapping or ‘sewing up’ of the region corresponding to the blind spot. Moreover, spatial alignment thresholds in healthy humans are lower across the blind spot than across intact retina (Crossland and Bex, 2009), consistent with involvement of a low-level mechanism in perceptual filling-in at the blind spot.

Single cell recordings from anaesthetized monkeys show that when filling-in takes place at the blind spot, neural responses are generated at the retinotopic representation of the blind spot in primary visual cortex (Fiorani et al., 1992, Matsumoto and Komatsu, 2005, Komatsu et al., 2002). Some V1 neurons activated during perceptual filling-in at the blind spot have large receptive fields, extending out of the blind spot (Komatsu et al., 2002), suggesting the passive importing of information from the surrounding visual field. Conversely, there is also strong evidence consistent for an active neural completion process as stronger activity is associated with bars spanning both sides of the blind spot than for bars stimulating only one side of the blind spot (Fiorani et al., 1992, Matsumoto and Komatsu, 2005). Moreover, this most recent study (Matsumoto and Komatsu, 2005) demonstrated response latencies in V1 that were 12 ms slower for stimuli presented to the blind spot eye than to the other eye. These response latencies did not increase when more distal regions of the receptive field were stimulated, suggesting that they were not due to long-range horizontal connections, but might instead reflect feedforward signals to V2 which then feedback to V1.

In humans, fMRI studies demonstrate the presence of a weakly responsive region in V1 corresponding to the cortical representation of the blind spot that is no longer evident in V2 (Tong and Engel, 2001, Tootell et al., 1998). A more recent study using fMRI (Awater et al., 2005) failed to find distortions of representation around the blind spot during stimulation of the two sides of the blind spot independently. This is consistent with neurophysiological studies showing that perceptual completion at the blind spot is likely to occur through an active completion mechanism (Fiorani et al., 1992, Matsumoto and Komatsu, 2005), although, crucially that fMRI study did not examine cortical representation around the blind spot during perceptual filling-in at the blind spot. Consistent with previous studies, they also showed that by V2, the gap in cortical representation corresponding to the blind spot was no longer present.

Taken together, these studies suggest that perceptual filling-in at the blind spot is likely to reflect active processes, probably comprising lateral propagation signals in primary visual cortex but also possibly involving feedback signals from extrastriate regions.

### 3.2 Filling-in across retinal scotomas

Patients with retinal scotomas due to macular degeneration or toxoplasma infection also experience perceptual filling-in (Gerrits and Timmerman, 1969, Zur and Ullman, 2003) which is instant, improves with increasing density and regularity of the filled-in patterns and occurs for scotomas as large as 6 degrees, at less than 2 mm from the fovea (Zur and Ullman, 2003). Retinal scotomas are also associated with a twinkle after-effect, for areas as large as 20 degrees (Crossland et al., 2007), consistent with an active completion process, but possibly in extrastriate areas, given the large area of this effect. Interestingly, alignment thresholds over pathological retinal scotomas are not lower than across intact retina (Crossland and Bex, 2009), suggesting that perceptual filling-in across retinal scotomas does not include low-level receptive field organization.

Single cell recordings in mammalian visual cortex have revealed conflicting results. In monkeys, cells within primary visual cortex representing the edge of the lesions expand their receptive fields within minutes after inducing bilateral retinal lesions (Gilbert and Wiesel, 1992), and several months after the lesion, the receptive fields have expanded and shifted to outside the lesion. Importantly, in adult mammals, this reorganization occurs within hours of the lesion (Chino et al., 1992), but only if associated with absence of input from the fellow eye. Similar reports of receptive field reorganization in V1 have been shown several weeks following cortical lesions in kittens (Zepeda et al., 2003) and in adult cats (Eysel and Schweigart, 1999). However, a recent report (Smirnakis et al., 2005) failed to show long-term reorganization in macaque V1 after bilateral retinal lesions. This discrepancy has proved controversial (Calford et al., 2005) but may be due to differences in the way measurements of cortical activity were made (see below).

Cortical reorganization might involve a sequence of mechanisms. The first stage involves expansion of receptive fields close to the lesion border, within minutes of the lesion, possibly due to unmasking of subthreshold excitatory inputs (Darian-Smith and Gilbert, 1994, Schweigart and Eysel, 2002). This might be followed by a more gradual phase of organization over the subsequent weeks and months, which may be mediated by axonal growth of cortical long-range horizontal collateral connections (Darian-Smith and Gilbert, 1994), or by reduction in inhibition producing re-emergent spiking activity in horizontal connections between normal and de-afferented cortex. This might explain the discrepancy between the earlier findings and those by Smirnakis and colleagues as changes following reorganization might involve augmentation of synapses producing re-emergent spiking activity, but not an anatomical restructuring. Smirnakis and colleagues measured only BOLD and multi-unit potentials, which are both more sensitive to synaptic input into a region, rather than the spiking output. Of note, this reorganization is restricted to cortical neurons as no reorganization occurs within LGN following retinal lesions in cats or monkeys (Darian-Smith and Gilbert, 1995).

In humans, reports are also inconsistent. Visual cortex (including V1) deprived of retinal input due to macular degeneration shows increased activation with functional MRI to stimuli outside the corresponding region in visual space (Baker et al., 2005, Baker et al., 2008). Reorganization also occurs following loss of visual input due to optic radiation damage following stroke (Dilks et al., 2007). However, another study failed to find consistent evidence for cortical reorganization in a single patient with macular degeneration (Sunness et al., 2004) and a further study in patients with juvenile macular degeneration found responses in visual cortex deprived of input only when patients were performing a stimulus-related task (and only in three out of four patients) (Masuda et al., 2008). How can these differences between studies in human patients be reconciled? Intriguingly, Sunness and colleagues measured responses during passive viewing of a checkerboard, whereas Baker and colleagues recorded activity during a stimulus-related task (similar to that used by Masuda and colleagues, although Baker et al. (2008) also found weak responses during passive viewing of the stimuli). Thus the task itself seems to be closely related to presence of activity in deprived cortex. Taking this into account, one possible mechanism might include formation of lateral connections that are insufficient to generate a BOLD response during passive viewing but are amplified by feedback signals from extrastriate cortex by task-related demands (but see Masuda et al., 2008). Alternatively, feedback signals from extrastriate cortex may be unmasked by the absence of signal in the deprived cortical regions. Importantly, both patient group studies found heterogeneity in responses, suggesting mechanisms may differ between individuals and a recent study suggests that large-scale cortical reorganization may only occur in association with the complete absence of functional foveal vision (Baker et al., 2008). Furthermore, significant neurochemical and structural modifications are likely to take place close to the lesion, particularly for cortical lesions, which may have important effects on local responses.

Finally, how do we explain the discrepancies between the reorganization following retinal damage in cat and monkey (Gilbert and Wiesel, 1992, Darian-Smith and Gilbert, 1994, Chino et al., 1992) with these findings in human patients? There are several possible explanations for these differences: the lesions in the neurophysiological experiments were much smaller, producing a deprived cortical region spanning up to 8 mm (Chino et al., 1992, Darian-Smith and Gilbert, 1994, Darian-Smith and Gilbert, 1995), compared to lesions of several centimeters in the human patients. Furthermore, the animal experiments were carried out on anesthetized animals and could therefore not examine the effect of feedback signals modulated by a task. Finally, it is possible that some small-scale reorganization of the type seen in cats and monkeys also takes place at the edges of deprived cortex but is unable to span the entire region.

## 4 Delayed perceptual filling-in, dependent on stimulus configuration

### 4.1 Troxler fading and artificial scotomas

Troxler fading refers to the tendency of stimuli placed in peripheral vision to gradually fade from view with maintained central fixation (Troxler, 1804) (Fig 4a, right panel). Stimuli are more likely to fade if they are peripheral and have indistinct edges (Friedman et al., 1999) and when the luminance (Sakaguchi, 2001) and contrast difference between the target and surround is reduced (Sakaguchi, 2006). The faded percept returns with eye movements or blinking and is counteracted by microsaccades (Martinez-Conde et al., 2006).

This perceptual filling-in can be made even more striking if the background is replaced by dynamic twinkling noise (Ramachandran and Gregory, 1991), which causes perceptual filling-in to occur more rapidly (Spillmann and Kurtenbach, 1992) and the percept is then termed an artificial scotoma (Ramachandran and Gregory, 1991) (see Fig. 4b).

The perceptual filling-in of artificial scotomas is similar in many ways to that which occurs with Troxler fading. Both require prolonged fixation before perceptual filling-in of the peripheral target can take place. Both require the target to be in the near periphery, and both processes are disrupted by eye movements and counteracted by microsaccades. Perceptual filling-in of artificial scotomas occurs earlier than for a target of equivalent size and eccentricity to fade, and it occurs more consistently for salient targets (e.g., red or flickering targets) than would occur for Troxler fading. Phenomenologically, a possible difference is that during perceptual filling-in of an artificial scotoma, the background is perceived at the position previously occupied by the target. Furthermore, researchers have argued that the filling-in of artificial scotomas is less likely to be due to adaptation as the border between the target and the surround is constantly refreshed (Spillmann and Kurtenbach, 1992). However, it is not entirely clear that these two phenomena are distinct and are not due to similar underlying neural processes.

Behavioral studies suggest that perceptual filling-in of artificial scotomas is associated with activity in early retinotopic cortex as filling-in is influenced by low-level sensory factors such as eccentricity and boundary length (De Weerd et al., 1998, Welchman and Harris, 2001) as well as increased differences in luminance or motion contrast between the target and its surround (Welchman and Harris, 2001). Similarly, the relative salience of the target compared to its background influences time to filling-in, with increasing perceptual salience associated with an increased fading time (Sturzel and Spillmann, 2001, Welchman and Harris, 2001).

Detection thresholds for Gabor patches presented within artificial scotomas are elevated by dynamic noise surrounds (Mihaylov et al., 2007), and after Troxler fading, participants are less able to detect a probe presented within a perceptually filled-in target (Lleras and Moore, 2006), suggesting suppression of target-associated signals during the subjective experience of perceptual completion. However, an earlier study (Kapadia et al., 1994) used a three-line bisection task to measure distortions around artificial scotomas and found that the apparent position of the middle line segment was drawn towards the interior of the artificial scotoma, suggesting possible reorganization around the perceptually filled-in target. This is also consistent with a more recent behavioral study suggesting cortical reorganization surrounding perceptually filled-in targets (Tailby and Metha, 2004).

Artificial scotomas can also induce after effects (Hardage and Tyler, 1995), consistent with an active process. Furthermore, high-level factors have also been shown to influence perceptual filling-in (De Weerd et al., 2006) as directing spatial attention to the peripheral target increases the probability of it perceptually filling-in. Taken together, these studies are consistent with perceptual filling-in of peripheral targets occurring in retinotopic visual cortex, possibly with some contribution from higher visual areas.

Physiological studies do not yet reveal a consistent pattern of neural activity associated with perceptual filling-in of artificial scotomas. In monkey, single neurons in V2 and V3 whose receptive fields overlap an achromatic target placed on dynamic noise increased their firing after a few seconds of eccentric fixation (De Weerd et al., 1995). However, it is not clear whether such changes correspond to perceptual completion as the monkeys did not report their perception and conversely, in responding monkeys, V1 and V2 boundary neurons show decreased activity during Troxler color filling-in (Von der Heydt et al., 2003).

In humans, luminance filling-in (filling-in of an achromatic target on a uniform achromatic background) is associated with a generalized (non-retinotopic) decrease in activation in V1 and V2 and increased activity in higher visual areas (Mendola et al., 2006). Similarly, target-specific responses to a perceptually filled-in artificial scotoma are reduced compared to responses to the visible target (Weil et al., 2007). However, even when invisible, target-specific responses remain, compared with a no-target baseline, indicating continued representation of these target-specific responses and consistent with an active process of perceptual filling-in. Neuroimaging studies reveal a locus for this process in the earliest cortical stages of the human visual pathways, in V1 and V2 (Weil et al., 2008). Taken together, these studies are consistent with perceptual filling-in of peripheral targets occurring in retinotopic visual cortex, with some contribution from higher visual areas.

### 4.2 Motion-induced blindness

Motion-induced blindness (MIB) is a striking phenomenon in which a perceptually salient target repeatedly disappears and then reappears when superimposed on a field of moving distractors, after a period of maintained central fixation (Bonneh et al., 2001) (Fig 4c). Target disappearance is influenced by low-level sensory factors such as eccentricity (Hsu et al., 2004) and size (Bonneh et al., 2001), the boundary adaptation effect (Hsu et al., 2006), and also placing the target behind the distractors (Graf et al., 2002).

However, several features of MIB distinguish it from other forms of peripheral perceptual filling-in such as Troxler fading and perceptual filling-in of artificial scotomas. Unlike peripheral perceptual filling-in, MIB targets perceptually fill-in more readily if they are of increased luminance and contrast compared to the background (Bonneh et al., 2001), although the luminance difference between the targets and the distractors should be small (Hsu et al., 2004) and MIB occurs after briefer periods of prolonged fixation. Furthermore, if MIB targets are surrounded by a region without any distractor targets, this region is spared from any perceptual filling-in.

MIB is unlikely to reflect local adaptation processes, as it persists for moving or flickering targets and even when targets and distracters are spatially separated (Bonneh et al., 2001). Furthermore, high-level factors seem to play an important role in MIB: disappearance of the target is subject to grouping effects, with targets tending to disappear together rather than separately if they form good gestalts (Bonneh et al., 2001), and when two Gabor patches are presented as targets, they disappear together if they are collinear and in alternation if their orientation is orthogonal (Bonneh et al., 2001). Even when targets are invisible, they can still generate orientation-specific after effects (Montaser-Kouhsari et al., 2004) or negative afterimages (Hofstoetter et al., 2004), suggesting that MIB occurs beyond the cortical site of these after effects. Finally, attended targets are more likely to disappear than unattended targets (Schölvinck and Rees, 2009).

Functional MRI studies have shown conflicting results. One study (Donner et al., 2008) found decreased activity associated with target disappearance in the region of V4 corresponding to the target but increased responses with target disappearance in regions of dorsal visual areas corresponding to the mask, particularly in Areas V3A and B and the posterior intraparietal sulcus. These responses were superimposed on a delayed and spatially non-specific reduction in response in visual Areas V1–3 during target disappearance. In contrast, a more recent study found that disappearance of the target was associated with increased activity in V1 and V2 regions corresponding to the target and in V5/MT contralateral to the target (Schölvinck and Rees, 2010) (although that more recent study did not examine responses in higher Areas V3A, V3B, or V4). Interestingly, both studies report a global response to target disappearance in early visual areas that is not specific to the target or the mask, although this response is opposite in direction, with a decrease in the earlier study and an increase in the more recent study. This may reflect a difference in the masks used (cloud of dots versus rotating grid), or possibly in the way the mask was treated in the analyses.

Interestingly, a recent study of motion-induced blindness in monkeys (Libedinsky et al., 2009) found a decrease in firing rate during target disappearance in V1 ON cells, but an increase in V1 OFF cells. This might explain the discrepancy in results between the human imaging studies, if different subpopulations of cells respond differently to the target disappearing. Ultimately, more work will be needed to determine the pattern of responses to MIB in visual cortex.

## 5 Delayed perceptual filling-in independent of stimulus configuration

### 5.1 Stabilized retinal images

When an image of an object is stabilized on the retina (for example, using an optical lever system to ensure that the stimulus moves opposite to eye movements to cancel out eye movements), after a few seconds, it gradually fades away and is replaced by the texture or color of the surrounding visual field (Ditchburn and Ginsborg, 1952). Notably, complete stabilization of the image (for example, by projecting the internal structure of the eye onto the retina) causes images to disappear and never return (Campbell and Robson, 1961). Within a stabilized image, single lines tend to disappear as units and parallel lines tend to disappear and reappear together. The length of time that the target remains visible is partly a function of the complexity of the target and meaningful figures remain visible for longer than meaningless figures (Pritchard et al., 1960). Studies have also shown features of interocular transfer (Krauskopf and Riggs, 1959) consistent with a cortical locus for the phenomenon. Moreover, the part of the image to which the participant is directing attention remains in view longer than other parts and stimulating other modalities such as a sudden noise causes reappearance of the image (Pritchard et al., 1960) consistent with involvement of higher regions in the disappearance of the images.

This phenomenon may be an adaptive mechanism to prevent retinal vessels from interrupting our view of the world and also demonstrates the importance of constant eye movements in continually stimulating the visual system and preventing the apparent disappearance of visual scenes. It is possible that other forms of perceptual filling-in following maintained fixation share some common mechanisms with the fading of stabilized retinal images.

## 6 Discussion

The framework described above is a new and potentially useful way of categorizing all forms of perceptual filling-in. By considering perceptual filling-in within these categories, the commonalities and differences between the various forms of filling-in become apparent, which is essential in considering the mechanisms underlying perceptual filling-in. Perceptual filling-in can be considered as an abnormal form of surface and contour perception, as contours or surfaces are perceived where they do not actually exist in visual space. It can be instant or delayed, and as becomes apparent from the framework described above, this depends on the presence or absence of existing boundaries. Where perceptual filling-in occurs in the absence of real boundaries, it will occur instantly. For example, if the perceptual completion involves the formation of new boundaries (as in illusory contours, where no boundary is present), or the spread of surface in the absence of a boundary (as in the blind spot), perceptual filling-in is instant.

Conversely, where perceptual filling-in occurs in the presence of an existing boundary, it will only occur after prolonged fixation, as this boundary must first be broken down by adaptive mechanisms, before perceptual filling-in can occur. For example, perceptual filling-in of artificial scotomas only occurs after prolonged fixation, as the boundary of the figure being filled-in must first be degraded by adaptive mechanisms. Similarly for Troxler fading, motion-induced blindness and stabilized retinal images.

This importance of boundaries in preventing interpolation processes has been discussed previously in the context of filling-in of artificial scotomas (De Weerd et al., 1995, Spillman and De Weerd, 2003), where a model has been proposed for a two-stage process involving adaptation of figure boundaries followed by a faster interpolation process where the background fills in the area previously occupied by the figure. However, the importance of boundaries has not previously been applied systematically to all forms of perceptual filling-in. By considering perceptual filling-in within this framework, the importance of boundary adaptation as a predictor of the timing of filling-in becomes immediately apparent.

### 6.1 Perceptual filling-in in the context of general perception of contours and surfaces

**Fig. 5:**
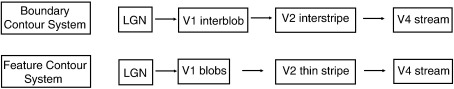
Schematic diagram of the Feature Contour System and the Boundary Contour System().

In order to understand perceptual filling-in, it is helpful to consider perceptual filling-in in the context of processes involved in general perception of contours and surfaces. One influential model of general perception is FACADE (Form-And-Color-And-DEpth) theory, described by Grossberg (1994). This theory describes two main systems in visual processing: the Boundary Contour System and the surface system, termed the Feature Contour System. According to the FACADE theory, all boundaries are in fact invisible and edges are only perceived as such as they arise as coherent patterns of excitatory and inhibitory signals across a feedback network from the retina through the LGN and V1 interblob and V2 interstripe areas (see Fig. 5). Long-range cooperative interactions build boundaries across space while interacting with shorter range inhibitory competitive interactions suppressing boundary groupings. These boundaries are, in a sense, invisible, as they are insensitive to contrast polarity, but simply pool information from cells sensitive to the same orientation but not necessarily of the same contrast polarities (Grossberg, 2003).

After Grossberg S: Filling-in the forms: Surface and boundary interactions in visual cortex. In Pessoa L, De Weerd P (eds): Filling-In: From Perceptual Completion to Cortical Reorganization. Oxford University Press, 2003, pp 13–37.

The surface system, on the other hand, termed the Feature Contour System, operates as a network from the retina, via the LGN to the V1 blob, V2 thin stripe and then V4 stream. It relies on discounting the illumination, to compensate for variable illumination and then filling-in the surface with color or brightness, in a form of diffusion across visual space. The surface system interacts with the Boundary Contour System to limit the diffusive spread of surface information. Conversely, the Boundary Contour System becomes visible through interactions with the surface system.

FACADE theory has been applied to perceptual filling-in previously (Grossberg, 2003). Here we extend it by applying it to the new system for categorizing perceptual filling-in presented here and use it to make predictions for all forms of perceptual filling-in. Where perceptual filling-in occurs for specific stimulus configurations following a delay, as in Troxler fading or filling-in of artificial scotomas, boundaries are invariably present. As discussed above and by others (De Weerd et al., 1998), in order for perceptual filling-in to take place, these boundaries must first be adapted, or broken down, thus explaining the initial delay.

Where filling-in occurs instantly but is dependent on stimulus configuration, as in illusory contours and neon color spreading, boundaries and surfaces are formed where they do not physically exist. This may explain why filling-in occurs instantly. Moreover, these illusions exploit specific feature configurations, which in the real world often signify borders and surfaces, such as in the context of camouflaged or occluded objects and are therefore interpreted by the visual system as the most likely natural configuration to explain the existing set of boundaries and surfaces.

It is likely that forms of perceptual filling-in that are dependent on stimulus configuration exploit common mechanisms to those underlying perceptual filling-in independent of stimulus configuration, which have been developed by the visual system to overcome deficits to visual system input. Thus, in instant filling-in independent of stimulus configuration, as in the filling-in that takes place across the blind spot, or across retinal scotomas, the Feature Contour System is not limited by any boundaries, so filling-in continues almost instantly across regions of visual space, possibly mediated by new horizontal connections, or shifts in the balance of inhibitory and excitatory inputs.

Conversely, retinal vessels do provide a boundary that would otherwise limit the spread of visual information. The visual system has therefore developed mechanisms to prevent such interruptions to vision by causing images stabilized on the retina to fade and disappear over time. These mechanisms are exploited in illusions such as Troxler fading and the disappearance of artificial scotomas.

## 7 Which model for perceptual filling-in?

Although perceptual filling-in takes many different forms and underlying processes are likely to differ in some respects, they may share some mechanisms. Most of the evidence presented here is consistent with an active process, in line with the isomorphic model of filling-in (Fiorani et al., 1992, Darian-Smith and Gilbert, 1994, Redies et al., 1986, Ohtani et al., 2002, Tong and Engel, 2001, Murakami, 1995), and many forms seem to involve the early stages of cortical processing, although this is often with some contribution from higher areas (De Weerd et al., 2006, Rauschenberger et al., 2006, Masuda et al., 2008). This is with the notable exception of evidence for the Craik–Cornsweet–O'Brien effect, which seems to be generated by amplification of image statistics at subcortical stages, more consistent with the symbolic theory.

The importance of boundaries that has emerged from this framework may form an intriguing area for future research. This might be specifically tested by generating new stimulus configurations that violate the framework presented here. For example, boundaries might be introduced into stimuli that usually generate illusory contours, with the aim of disrupting or slowing down the usually instant process. Alternatively, it would be interesting to attempt to speed up delayed forms of perceptual filling-in by removing boundaries. For example, the time to filling-in of real and illusory contours could be compared in the context of motion-induced blindness.

## 8 Conclusion

Here we have presented a new framework for categorizing the different forms of perceptual filling-in. We have reviewed the literature for each different form of perceptual filling-in within the context of this new framework, and we have considered how this framework highlights commonalities and differences between forms of perceptual filling-in within the context of a classic model of surface and boundary perception. Finally, we have considered the importance of the presence or absence of boundaries in determining the latency of different forms of perceptual filling-in.

## References

[bb0005] Anderson B.L., Singh M., Fleming R.W. (2002). The interpolation of object and surface structure. Cogn Psychol.

[bb0010] Anderson E.J., Dakin S.C., Rees G. (2009). Monocular signals in human lateral geniculate nucleus reflect the Craik–Cornsweet–O'Brien effect. J Vis.

[bb0015] Awater H., Kerlin J.R., Evans K.K., Tong F. (2005). Cortical representation of space around the blind spot. J Neurophysiol.

[bb0020] Baker C.I., Peli E., Knouf N., Kanwisher N.G. (2005). Reorganization of visual processing in macular degeneration. J Neurosci.

[bb0025] Baker C.I., Dilks D.D., Peli E., Kanwisher N. (2008). Reorganization of visual processing in macular degeneration: replication and clues about the role of foveal loss. Vision Res.

[bb0030] Bakin J.S., Nakayama K., Gilbert C.D. (2000). Visual responses in monkey Areas V1 and V2 to three-dimensional surface configurations. J Neurosci.

[bb0035] Berkley M.A., Debruyn B., Orban G. (1994). Illusory, motion, and luminance-defined contours interact in the human visual system. Vision Res.

[bb0040] Bonneh Y.S., Cooperman A., Sagi D. (2001). Motion-induced blindness in normal observers. Nature.

[bb0045] Boselie F., Wouterlood D. (1992). A critical discussion of Kellman and Shipley's (1991) theory of occlusion phenomena. Psychol Res.

[bb0050] Bressan P., Mingolla E., Spillmann L., Watanabe T. (1997). Neon color spreading: a review. Perception.

[bb0055] Buffart H., Leeuwenberg E. (1981). Coding theory of visual pattern completion. J Exp Psychol Hum Percept Perform.

[bb0060] Burr D.C. (1987). Implications of the Craik–O'Brien illusion for brightness perception. Vision Res.

[bb0065] Calford M.B., Chino Y.M., Das A., Eysel U.T., Gilbert C.D., Heinen S.J., Kaas J.H., Ullman S. (2005). Neuroscience: rewiring the adult brain. Nature.

[bb0070] Campbell F.W., Robson J.G. (1961). A fresh approach to stabilised retinal images. J Physiol.

[bb0075] Campbell F.W., Howell E.R., Johnstone J.R. (1978). A comparison of threshold and suprathreshold appearance of gratings with components in the low and high spatial frequency range. J Physiol.

[bb0080] Chino Y.M., Kaas J.H., Smith E.L., Langston A.L., Cheng H. (1992). Rapid reorganization of cortical maps in adult cats following restricted deafferentation in retina. Vision Res.

[bb0085] Chino Y., Smith E.L., Zhang B., Matsuura K., Mori T., Kaas J.H. (2001). Recovery of binocular responses by cortical neurons after early monocular lesions. Nat Neurosci.

[bb0090] Cohen M.A., Grossberg S. (1984). Neural dynamics of brightness perception: features, boundaries, diffusion, and resonance. Percept Psychophys.

[bb0095] Cornsweet T. (1970).

[bb0100] Craik K.J.W. (1966). The Nature of Psychology.

[bb0105] Crossland M.D., Bex P.J. (2009). Spatial alignment over retinal scotomas. Invest Ophthalmol Vis Sci.

[bb0110] Crossland M.D., Dakin S.C., Bex P.J. (2007). Illusory stimuli can be used to identify retinal blind spots. PLoS ONE.

[bb0115] Dakin S.C., Bex P.J. (2003). Natural image statistics mediate brightness ‘filling in’. Proc Biol Sci.

[bb0120] Darian-Smith C., Gilbert C.D. (1994). Axonal sprouting accompanies functional reorganization in adult cat striate cortex. Nature.

[bb0125] Darian-Smith C., Gilbert C.D. (1995). Topographic reorganization in the striate cortex of the adult cat and monkey is cortically mediated. J Neurosci.

[bb0130] Davey M.P., Maddess T., Srinivasan M.V. (1998). The spatiotemporal properties of the Craik–O'Brien–Cornsweet effect are consistent with ‘filling-in’. Vision Res.

[bb0135] Davis G., Driver J. (1994). Parallel detection of Kanizsa subjective figures in the human visual system. Nature.

[bb0140] Davis G., Driver J., Pessoa L., De Weerd P. (2003). Filling-in. From perceptual completion to cortical reorganization.

[bb0145] De Weerd P. (2006). Perceptual filling-in: more than the eye can see. Prog Brain Res.

[bb0150] De Weerd P., Gattass R., Desimone R., Ungerleider L.G. (1995). Responses of cells in monkey visual cortex during perceptual filling-in of an artificial scotoma. Nature.

[bb0155] De Weerd P., Desimone R., Ungerleider L.G. (1998). Perceptual filling-in: a parametric study. Vision Res.

[bb0160] De Weerd P., Smith E., Greenberg P. (2006). Effects of selective attention on perceptual filling-in. J Cogn Neurosci.

[bb0165] Dennett D. (1991).

[bb0170] Devinck F., Hansen T., Gegenfurtner K.R. (2007). Temporal properties of the chromatic and achromatic Craik–O'Brien–Cornsweet effect. Vision Res.

[bb0175] Dilks D.D., Serences J.T., Rosenau B.J., Yantis S., McCloskey M. (2007). Human adult cortical reorganization and consequent visual distortion. J Neurosci.

[bb0180] Ditchburn R.W., Ginsborg B.L. (1952). Vision with a stabilized retinal image. Nature.

[bb0185] Donner T.H., Sagi D., Bonneh Y.S., Heeger D.J. (2008). Opposite neural signatures of motion-induced blindness in human dorsal and ventral visual cortex. J Neurosci.

[bb0190] Driver J., Davis G., Russell C., Turatto M., Freeman E. (2001). Segmentation, attention and phenomenal visual objects. Cognition.

[bb0195] Eysel U.T., Schweigart G. (1999). Increased receptive field size in the surround of chronic lesions in the adult cat visual cortex. Cereb Cortex.

[bb0200] Fiorani J.M., Rosa M.G., Gattass R., Rocha-Miranda C.E. (1992). Dynamic surrounds of receptive fields in primate striate cortex: a physiological basis for perceptual completion?. Proc Natl Acad Sci USA.

[bb0205] Friedman H.S., Zhou H., Von der Heydt H.R. (1999). Color filling-in under steady fixation: behavioral demonstration in monkeys and humans. Perception.

[bb0210] Gerrits H.J., Timmerman G.J. (1969). The filling-in process in patients with retinal scotomata. Vision Res.

[bb0215] Gilbert C.D., Wiesel T.N. (1992). Receptive field dynamics in adult primary visual cortex. Nature.

[bb0220] Graf E.W., Adams W.J., Lages M. (2002). Modulating motion-induced blindness with depth ordering and surface completion. Vision Res.

[bb0225] Grossberg S. (1994). 3-D vision and figure-ground separation by visual cortex. Percept Psychophys.

[bb0230] Grossberg S., Pessoa L., De Weerd P. (2003). Filling-in: From Perceptual Completion to Cortical Reorganization.

[bb0235] Halgren E., Mendola J., Chong C.D., Dale A.M. (2003). Cortical activation to illusory shapes as measured with magnetoencephalography. Neuroimage.

[bb0240] Hardage L., Tyler C.W. (1995). Induced twinkle aftereffect as a probe of dynamic visual processing mechanisms. Vision Res.

[bb0245] Hegde J., Fang F., Murray S.O., Kersten D. (2008). Preferential responses to occluded objects in the human visual cortex. J Vis.

[bb0250] Hirsch J., DeLaPaz R.L., Relkin N.R., Victor J., Kim K., Li T., Borden P., Rubin N., Shapley R. (1995). Illusory contours activate specific regions in human visual cortex: evidence from functional magnetic resonance imaging. Proc Natl Acad Sci USA.

[bb0255] Hofstoetter C., Koch C., Kiper D.C. (2004). Motion-induced blindness does not affect the formation of negative afterimages. Conscious Cogn.

[bb0260] Hsu L.C., Yeh S.L., Kramer P. (2004). Linking motion-induced blindness to perceptual filling-in. Vision Res.

[bb0265] Hsu L.C., Yeh S.L., Kramer P. (2006). A common mechanism for perceptual filling-in and motion-induced blindness. Vision Res.

[bb0270] Hung C.P., Ramsden B.M., Chen L.M., Roe A.W. (2001). Building surfaces from borders in Areas 17 and 18 of the cat. Vision Res.

[bb0275] Huxlin K.R., Saunders R.C., Marchionini D., Pham H.A., Merigan W.H. (2000). Perceptual deficits after lesions of inferotemporal cortex in macaques. Cereb Cortex.

[bb0280] Johnson J.S., Olshausen B.A. (2005). The recognition of partially visible natural objects in the presence and absence of their occluders. Vision Res.

[bb0285] Kanizsa G. (1979).

[bb0290] Kapadia M.K., Gilbert C.D., Westheimer G. (1994). A quantitative measure for short-term cortical plasticity in human vision. J Neurosci.

[bb0295] Kellman P.J., Shipley T.F. (1991). A theory of visual interpolation in object perception. Cogn Psychol.

[bb0300] Kingdom F., Moulden B. (1988). Border effects on brightness: a review of findings, models and issues. Spat Vis.

[bb0305] Komatsu H. (2006). The neural mechanisms of perceptual filling-in. Nat Rev Neurosci.

[bb0310] Komatsu H., Kinoshita M., Murakami I. (2002). Neural responses in the primary visual cortex of the monkey during perceptual filling-in at the blind spot. Neurosci Res.

[bb0315] Krauskopf J., Riggs L.A. (1959). Interocular transfer in the disappearance of stabilised images. Am J Psychol.

[bb0320] Kruggel F., Herrmann C.S., Wiggins C.J., von Cramon D.Y. (2001). Hemodynamic and electroencephalographic responses to illusory figures: recording of the evoked potentials during functional MRI. Neuroimage.

[bb0325] Larsson J., Amunts K., Gulyas B., Malikovic A., Zilles K., Roland P.E. (1999). Neuronal correlates of real and illusory contour perception: functional anatomy with PET. Eur J Neurosci.

[bb0330] Lee T.S., Nguyen M. (2001). Dynamics of subjective contour formation in the early visual cortex. Proc Natl Acad Sci USA.

[bb0335] Lerner Y., Hendler T., Malach R. (2002). Object-completion effects in the human lateral occipital complex. Cereb Cortex.

[bb0340] Lerner Y., Harel M., Malach R. (2004). Rapid completion effects in human high-order visual areas. Neuroimage.

[bb0345] Libedinsky C., Savage T., Livingstone M. (2009). Perceptual and physiological evidence for a role for early visual areas in motion-induced blindness. J Vis.

[bb0350] Lleras A., Moore C.M. (2006). What you see is what you get: functional equivalence of a perceptually filled-in surface and a physically presented stimulus. Psychol Sci.

[bb0355] Maertens M., Pollmann S., Hanke M., Mildner T., Moller H. (2008). Retinotopic activation in response to subjective contours in primary visual cortex. Front Hum Neurosci.

[bb0360] Martinez-Conde S., Macknik S.L., Troncoso X.G., Dyar T.A. (2006). Microsaccades counteract visual fading during fixation. Neuron.

[bb0365] Masuda Y., Dumoulin S.O., Nakadomari S., Wandell B.A. (2008). V1 projection zone signals in human macular degeneration depend on task, not stimulus. Cereb Cortex.

[bb0370] Matsumoto M., Komatsu H. (2005). Neural responses in the macaque v1 to bar stimuli with various lengths presented on the blind spot. J Neurophysiol.

[bb0375] Mattingley J.B., Davis G., Driver J. (1997). Preattentive filling-in of visual surfaces in parietal extinction. Science.

[bb0380] Mendola J.D., Dale A.M., Fischl B., Liu A.K., Tootell R.B. (1999). The representation of illusory and real contours in human cortical visual areas revealed by functional magnetic resonance imaging. J Neurosci.

[bb0385] Mendola J.D., Conner I.P., Sharma S., Bahekar A., Lemieux S. (2006). fMRI measures of perceptual filling-in in the human visual cortex. J Cogn Neurosci.

[bb0390] Michotte A., Thines G., Crabbe G., Thines G., Costall A., Butterworth G. (1991). Michotte's Experimental Phenomonelogy of Perception.

[bb0395] Mihaylov P., Manahilov V., Simpson W.A., Strang N.C. (2007). Induced internal noise in perceptual artificial scotomas created by surrounding dynamic noise. Vision Res.

[bb0400] Montaser-Kouhsari L., Moradi F., Zandvakili A., Esteky H. (2004). Orientation-selective adaptation during motion-induced blindness. Perception.

[bb0405] Montaser-Kouhsari L., Landy M.S., Heeger D.J., Larsson J. (2007). Orientation-selective adaptation to illusory contours in human visual cortex. J Neurosci.

[bb0410] Murakami I. (1995). Motion aftereffect after monocular adaptation to filled-in motion at the blind spot. Vision Res.

[bb0415] Murray M.M., Wylie G.R., Higgins B.A., Javitt D.C., Schroeder C.E., Foxe J.J. (2002). The spatiotemporal dynamics of illusory contour processing: combined high-density electrical mapping, source analysis, and functional magnetic resonance imaging. J Neurosci.

[bb0420] Murray M.M., Foxe D.M., Javitt D.C., Foxe J.J. (2004). Setting boundaries: brain dynamics of modal and amodal illusory shape completion in humans. J Neurosci.

[bb0425] Nakayama K., Shimojo S. (1992). Experiencing and perceiving visual surfaces. Science.

[bb0430] Nakayama K., Shimojo S., Silverman G.H. (1989). Stereoscopic depth: its relation to image segmentation, grouping, and the recognition of occluded objects. Perception.

[bb0435] Nakayama K., Shimojo S., Ramachandran V.S. (1990). Transparency: relation to depth, subjective contours, luminance, and neon color spreading. Perception.

[bb0440] Neumann H., Pessoa L., Hansen T. (2001). Visual filling-in for computing perceptual surface properties. Biol Cybern.

[bb0445] Nieder A. (2002). Seeing more than meets the eye: processing of illusory contours in animals. J Comp Physiol A Neuroethol Sens Neural Behav Physiol.

[bb0450] O'Brien V. (1958). Contour perception, illusion and reality. J Opt Soc Am.

[bb0455] Ohtani Y., Okamura S., Shibasaki T., Arakawa A., Yoshida Y., Toyama K., Ejima Y. (2002). Magnetic responses of human visual cortex to illusory contours. Neurosci Lett.

[bb0460] O'Regan J.K. (1992). Solving the “real” mysteries of visual perception: the world as an outside memory. Can J Psychol.

[bb0465] Perna A., Tosetti M., Montanaro D., Morrone M.C. (2005). Neuronal mechanisms for illusory brightness perception in humans. Neuron.

[bb0470] Pessoa L., Thompson E., Noe A. (1998). Finding out about filling-in: a guide to perceptual completion for visual science and the philosophy of perception. Behav Brain Sci.

[bb0475] Peterhans E., von der Heydt H.R. (1989). Mechanisms of contour perception in monkey visual cortex. II. Contours bridging gaps. J Neurosci.

[bb0480] Pillow J., Rubin N. (2002). Perceptual completion across the vertical meridian and the role of early visual cortex. Neuron.

[bb0485] Pinna B., Grossberg S. (2005). The watercolor illusion and neon color spreading: a unified analysis of new cases and neural mechanisms. J Opt Soc Am A Opt Image Sci Vis.

[bb0490] Pritchard R.M., Heron W., Hebb D.O. (1960). Visual perception approached by the method of stabilized images. Can J Psychol.

[bb0495] Proverbio A.M., Zani A. (2002). Electrophysiological indexes of illusory contours perception in humans. Neuropsychologia.

[bb0500] Ramachandran V.S. (1992). Filling in the blind spot. Nature.

[bb0505] Ramachandran V.S., Gregory R.L. (1991). Perceptual filling in of artificially induced scotomas in human vision. Nature.

[bb0510] Ramachandran V.S., Ruskin D., Cobb S., Rogers-Ramachandran D., Tyler C.W. (1994). On the perception of illusory contours. Vision Res.

[bb0515] Rauschenberger R., Liu T., Slotnick S.D., Yantis S. (2006). Temporally unfolding neural representation of pictorial occlusion. Psychol Sci.

[bb0520] Redies C., Spillman L. (1982). The neon color effect in the Ehrenstein illusion. Perception.

[bb0525] Redies C., Crook J.M., Creutzfeldt O.D. (1986). Neuronal responses to borders with and without luminance gradients in cat visual cortex and dorsal lateral geniculate nucleus. Exp Brain Res.

[bb0530] Rensink R.A., Enns J.T. (1998). Early completion of occluded objects. Vision Res.

[bb0535] Roe A.W., Lu H.D., Hung C.P. (2005). Cortical processing of a brightness illusion. Proc Natl Acad Sci USA.

[bb0540] Sakaguchi Y. (2001). Target/surround asymmetry in perceptual filling-in. Vision Res.

[bb0545] Sakaguchi Y. (2006). Contrast dependency in perceptual filling-in. Vision Res.

[bb0550] Sary G., Chadaide Z., Tompa T., Koteles K., Kovacs G., Benedek G. (2007). Illusory shape representation in the monkey inferior temporal cortex. Eur J Neurosci.

[bb0555] Sasaki Y., Watanabe T. (2004). The primary visual cortex fills in color. Proc Natl Acad Sci USA.

[bb0560] Schölvinck M.L., Rees G. (2009). Attentional influences on the dynamics of motion-induced blindness. J Vis.

[bb0565] Schölvinck M.L., Rees G. (2010). Neural correlates of motion-induced blindness in the human brain. J Cogn Neurosci.

[bb0570] Schweigart G., Eysel U.T. (2002). Activity-dependent receptive field changes in the surround of adult cat visual cortex lesions. Eur J Neurosci.

[bb0575] Seghier M.L., Vuilleumier P. (2006). Functional neuroimaging findings on the human perception of illusory contours. Neurosci Biobehav Rev.

[bb0580] Seghier M., Dojat M., Delon-Martin C., Rubin C., Warnking J., Segebarth C., Bullier J. (2000). Moving illusory contours activate primary visual cortex: an fMRI study. Cereb Cortex.

[bb0585] Sergent J. (1988). An investigation into perceptual completion in blind areas of the visual field. Brain.

[bb0590] Sheth B.R., Sharma J., Rao S.C., Sur M. (1996). Orientation maps of subjective contours in visual cortex. Science.

[bb0595] Smirnakis S.M., Brewer A.A., Schmid M.C., Tolias A.S., Schuz A., Augath M., Inhoffen W., Wandell B.A., Logothetis N.K. (2005). Lack of long-term cortical reorganization after macaque retinal lesions. Nature.

[bb0600] Smith A., Over R. (1975). Tilt aftereffects with subjective contours. Nature.

[bb0605] Spillman L., De Weerd P., Pessoa L., De Weerd P. (2003). Filling-in: From Perceptual Completion to Cortical Reorganisation.

[bb0610] Spillmann L., Kurtenbach A. (1992). Dynamic noise backgrounds facilitate target fading. Vision Res.

[bb0615] Spillmann L., Otte T., Hamburger K., Magnussen S. (2006). Perceptual filling-in from the edge of the blind spot. Vision Res.

[bb0620] Stanley D.A., Rubin N. (2003). fMRI activation in response to illusory contours and salient regions in the human lateral occipital complex. Neuron.

[bb0625] Sturzel F., Spillmann L. (2001). Texture fading correlates with stimulus salience. Vision Res.

[bb0630] Sugita Y. (1999). Grouping of image fragments in primary visual cortex. Nature.

[bb0635] Sunness J.S., Liu T., Yantis S. (2004). Retinotopic mapping of the visual cortex using functional magnetic resonance imaging in a patient with central scotomas from atrophic macular degeneration. Ophthalmology.

[bb0640] Tailby C., Metha A. (2004). Artificial scotoma-induced perceptual distortions are orientation dependent and short lived. Vis Neurosci.

[bb0645] Takeichi H., Shimojo S., Watanabe T. (1992). Neon flank and illusory contour: interaction between the two processes leads to color filling-in. Perception.

[bb0650] Tong F., Engel S.A. (2001). Interocular rivalry revealed in the human cortical blind-spot representation. Nature.

[bb0655] Tootell R.B., Mendola J.D., Hadjikhani N.K., Liu A.K., Dale A.M. (1998). The representation of the ipsilateral visual field in human cerebral cortex. Proc Natl Acad Sci USA.

[bb0660] Tripathy S.P., Levi D.M., Ogmen H. (1996). Two-dot alignment across the physiological blind spot. Vision Res.

[bb0665] Troxler D. (1804). Ophthalmische Bibliothek.

[bb0670] Tse P.U. (1999). Volume completion. Cogn Psychol.

[bb0675] van Lier R., van der H.P., Leeuwenberg E. (1994). Integrating global and local aspects of visual occlusion. Perception.

[bb0680] van Lier R., Vergeer M., Anstis S. (2009). Filling-in afterimage colors between the lines. Curr Biol.

[bb0685] van Tuijl H.F. (1975). A new visual illusion: neonlike color spreading and complementary color induction between subjective contours. Acta Psychol Amst.

[bb0690] Varin D. (1971). Fenomeni di contrasto e diffusione cromatica nell'organizzazione spaziale del campo percettivo. Riv Psicol.

[bb0695] Von der Heydt R., Peterhans E. (1989). Mechanisms of contour perception in monkey visual cortex. I. Lines of pattern discontinuity. J Neurosci.

[bb0700] Von der Heydt R., Friedman H., Zhou H., Pessoa L., De Weerd P. (2003). Filling-in. From Perceptual Completion to Cortical Reorganisation.

[bb0705] Watanabe T., Takeichi H. (1990). The relation between color spreading and illusory contours. Percept Psychophys.

[bb0710] Weigelt S., Singer W., Muckli L. (2007). Separate cortical stages in amodal completion revealed by functional magnetic resonance adaptation. BMC Neurosci.

[bb0715] Weil R.S., Kilner J.M., Haynes J.D., Rees G. (2007). Neural correlates of perceptual filling-in of an artificial scotoma in humans. Proc Natl Acad Sci USA.

[bb0720] Weil R.S., Watkins S., Rees G. (2008). Neural correlates of perceptual completion of an artificial scotoma in human visual cortex measured using functional MRI. Neuroimage.

[bb0725] Welchman A.E., Harris J.M. (2001). Filling-in the details on perceptual fading. Vision Res.

[bb0730] Zepeda A., Vaca L., Arias C., Sengpiel F. (2003). Reorganization of visual cortical maps after focal ischemic lesions. J Cereb Blood Flow Metab.

[bb0735] Zur D., Ullman S. (2003). Filling-in of retinal scotomas. Vision Res.

